# Periorbital skin index as a biomarker for biological aging and health status

**DOI:** 10.3389/fragi.2026.1715245

**Published:** 2026-01-29

**Authors:** Ki-Nam Gu, Sangseob Leem, Hanji Kim, Joong-Gon Shin, Jung Yeon Seo, Sunghwan Hwang, Eui Taek Jeong, Yunkwan Kim, Nae Gyu Kang

**Affiliations:** Research and Innovation Institute, R&D Center, LG H&H, Seoul, Republic of Korea

**Keywords:** periorbital skin age, wrinkles, pigmented spots, morphology, biological age, medical history

## Abstract

**Background:**

The periorbital skin area is particularly susceptible to aging compared to other facial regions due to its unique anatomical features and frequent muscle movements. This leads to early development of wrinkles and discoloration, which affect one’s appearance. Because of these characteristics, the eye-region skin serves as a representative indicator reflecting both skin aging and overall health status.

**Objectives:**

This study aims to develop and validate a straightforward, non-invasive method to evaluate changes in the eye-region skin as reliable markers of aging and overall physiological condition.

**Methods:**

We analyzed facial images from 2,515 Korean women aged 20–69 and evaluated various periorbital features, including wrinkles, morphological characteristics, and pigmented spots, using skin measurement devices and computational image analysis techniques. To assess skin aging and health status, we developed age prediction models based on different combinations of these periorbital features for each individual. Subsequently, Principal Component Analysis (PCA) was performed to summarize disease history variables for each participant, and the correlation between the first principal component (PC1) and periorbital skin age was evaluated using Pearson correlation analysis.

**Results:**

Periorbital skin features showed significant associations with chronological age. We developed nine distinct age prediction models by combining different subsets of these features, each producing a unique aging score. Among them, seven models demonstrated strong correlations with actual age (r > 0.7), confirming their predictive reliability. These individual model outputs were collectively considered as unified aging markers representing periorbital skin age. To evaluate clinical relevance, we analyzed the association between periorbital skin age derived from the model incorporating all skin features and disease history. Periorbital skin age showed significant associations with five out of seven diseases individually, as well as with the PC1 summarizing all disease histories collectively.

**Conclusion:**

This study establishes ‘periorbital skin age’ as a non-invasive biomarker that effectively reflects both the progression of skin aging and underlying medical conditions. Our findings highlight the potential utility of eye-region skin assessment in clinical and health monitoring settings, offering a practical tool for evaluating physiological aging and disease risk.

## 1 Introduction

The periorbital area is especially susceptible to aging compared to other facial areas due to its distinct anatomical and physiological features (Takema et al., 1997; Colvan et al., 2019). The region is encircled by the orbicularis oculi muscle, which is responsible for blinking and facial expressions. The repeated skin flexures resulting from facial muscle movements around the eyes contribute to the development of periorbital wrinkles (Lambros, 2007; Fujimura and Hotta, 2012). The periorbital skin is thinner than the other areas of facial skin, and contains fewer sweat and sebaceous glands, which reduces its capacity to retain moisture and weakens barrier function (Ha et al., 2005). Such vulnerability makes the periorbital region more prone to aging, resulting in the development of dark circles, fine lines, and wrinkles (Sarkar et al., 2016).

Diverse studies have investigated the aging characteristics of periorbital skin. Age-related changes in periorbital morphology include eyelid and lateral canthus sagging, as well as a reduction in the inter-canthal distance (Lee et al., 2019; Chong et al., 2021). Crow’s feet are typically less pronounced in younger individuals but tend to progress more rapidly than under-eye wrinkles (Tsukahara et al., 2013). Another study discovered a strong correlation of more than 80% between the severity of sagging eyelids and wrinkles around the eyes and perceived age (Flament et al., 2021). Given that people’s initial impressions of an individual can be greatly influenced by the appearance of the periorbital region (Wolffhechel et al., 2014; Ganel and Goodale, 2021), its aging process not only has clinical and social implications but may also serve as a useful biomarker for reflecting overall physiological aging.

While chronological age has long served as a traditional indicator of aging, it often fails to capture the heterogeneity of physiological decline. To address this limitation, biological age has been proposed as a more comprehensive and accurate measure of health status. Conventional biomarkers of biological age, such as telomere length and epigenetic modifications have been extensively studied (Levine et al., 2018; Fasching, 2018). More recently, novel approaches, including microbiome profiling and exosome composition analysis, have emerged as promising tools for assessing biological age and diagnosing age-related diseases (Mathur et al., 2024). However, the measurement of these markers still faces practical challenges, as most require invasive sampling and complex molecular analyses.

In light of these limitations, increasing attention has been directed toward simpler and less invasive biomarkers. For example, a large-scale study examining perceived youthfulness as a biomarker demonstrated associations with clinical history and genetic effects, suggesting the potential value of externally observable traits (Rippon and Steptoe, 2015; Ingold et al., 2024). Despite these attempts, studies exploring specific facial areas or components as independent biological age indicators remain limited. Therefore, we focused our investigation on periorbital skin aging as a potential aging biomarker, given its ability to effectively represent overall skin aging patterns.

In this study, we developed a novel approach to quantify periorbital skin aging and explored its potential as a biomarker for reflecting aging and individual medical history. Previous evidence suggests that molecular mechanisms involved in skin aging are closely connected to systemic aging and broader health status (Ghonemy et al., 2021; Miyawaki et al., 2016; López-Otín et al., 2013; Ferrucci and Fabbri, 2018; Olivieri et al., 2018). We collected front-facing photographs, medical history, and various skin characteristics from 2,515 participants and examined how facial features can reflect aging and medical history. Through the integration of the periorbital skin features, we developed nine age prediction models resulting in a single biomarker termed ‘periorbital skin age’. By creating this unified periorbital skin age measure, we present a novel approach to facial aging and physiological health.

## 2 Materials and Methods

### 2.1 Study design overview

**FIGURE 1:**
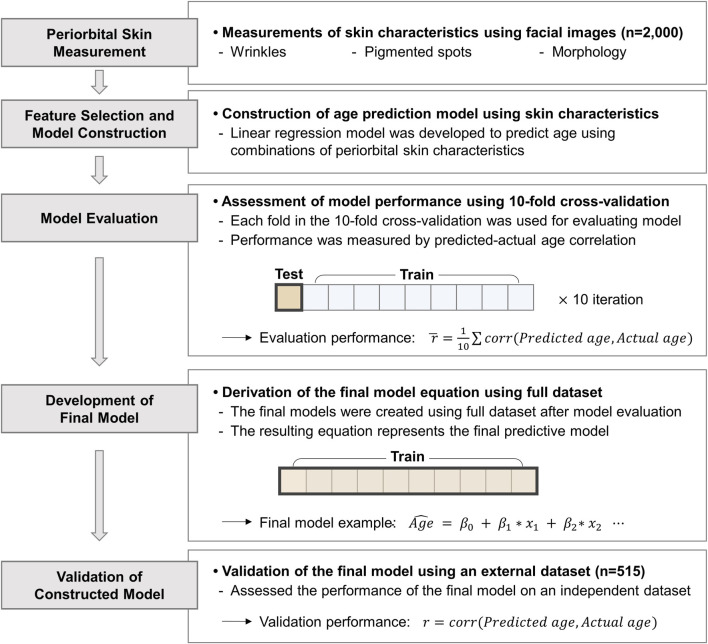
Overview of the study design. Periorbital skin characteristics were used to develop linear regression models for age estimation. The models were evaluated using 10-fold cross-validation. Final models were derived using the full dataset and validated with an independent dataset of 515 participants. *corr*, Pearson correlation coefficient; $\hat{Age}$ *,* Estimated age; β , Beta coefficient; x , Skin characteristics.

The study design, illustrated in Figure 1, involved collecting front-facing images from 2,000 participants and measuring three periorbital skin characteristics: wrinkles, pigmented spots, and morphology. These characteristics were used as independent variables in linear regression models to estimate participants’ ages. Multiple age prediction models were constructed using the combinations of these periorbital skin characteristics. The models were evaluated using 10-fold cross-validation, splitting the data into 90% training and 10% testing sets. Evaluation performance was assessed by averaging the correlation coefficients between predicted and actual ages across all 10 folds for each model. Following performance evaluation, final model equations were derived using the full dataset, and were validated using an independent dataset of 515 participants. The final model equations were used to estimate predicted ages, which we termed ‘periorbital skin age’, reflecting individual aging and health record.

### 2.2 Study subjects

We randomly selected a total of 2,515 Korean women from our previous study (Kim et al., 2024). Initially, we used data from 2,000 individuals aged 20–69 years, with each 10-year-old interval consisting of 400 people to construct the models. Subsequently, we used data from an additional 515 participants for the validation set. This validation set included 103 individuals for each 10-year interval. Demographic information including sex, age, sun exposure duration, frequency of sunscreen application, and smoking status, and medical history including previous disease diagnoses was collected through a survey questionnaire. Disease history was self-reported by participants who selected all applicable conditions they have ever experienced or been diagnosed with from a predefined list, including kidney disease, heart disease, hypertension, diabetes, hyperlipidemia, fatty liver, cancer, and none. This study was approved by the institutional review board (IRB) at the LG H&H Research Center (Seoul, South Korea) (IRB No. 2017-PB-0001 and LGHH-20180727-AA-03). The research was conducted in accordance with the Bioethics and Safety Act of South Korea. All participants were fully informed about this study and asked to sign an IRB-approved written consent form.

### 2.3 Measurements of two skin aging characteristics around the eyes

**FIGURE 2:**
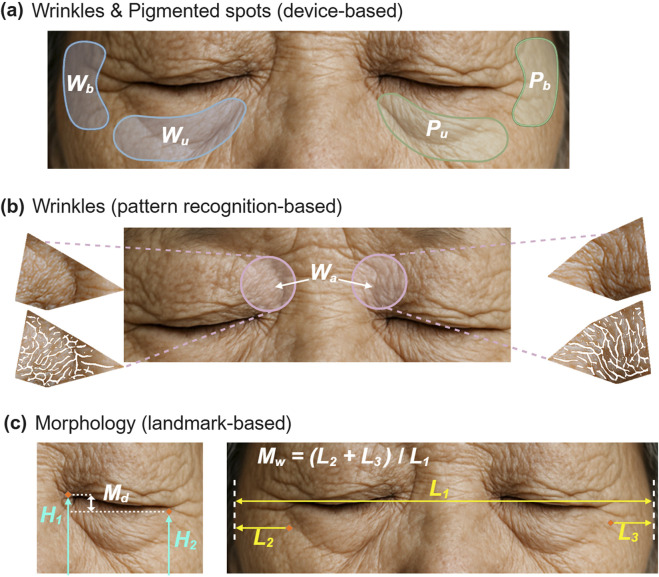
Aging characteristics of periorbital skin. **(a)** Detection of wrinkles and pigmented spots surrounding the eyes with the Janus-III skin measurement device: Wrinkles beside the eyes ( $W_{b}$ ), wrinkles under the eyes ( $W_{u}$ ), pigmented spots beside the eyes ( $P_{b}$ ), pigmented spots under the eyes ( $P_{u}$ ). Each set of 4 indices was measured in both the left and right regions. **(b)** Wrinkles above the inner corner of the eyes ( $W_{a}$ ) extracted by pattern recognition-based method. **(c)** Eye drooping ( $M_{d}$ ) in the left panel and temporal width ( $M_{w}$ ) in the right panel calculated using facial landmarks. *The face image included in this figure was generated using artificial intelligence*.

After the subjects washed their faces and waited indoors, we captured their facial images using a high-resolution camera-based skin measurement system (Janus-III, Korea, Suwon) with Canon 200D DSLR cameras (Canon, Tokyo, Japan) (Leem et al., 2020; Leem et al., 2021). The built-in algorithm of the system detected four periorbital areas considering the left and right regions, as depicted in Figure 2a, including the areas beside and under the eyes. If any abnormal regions were identified, manual adjustments were conducted. The algorithm quantified the degree of wrinkles and pigmented spots in the designated areas, generating values ranging from 0 to 100. We labeled the wrinkles beside the eyes as $W_{b}$ , the wrinkles under the eyes as $W_{u}$ , the pigmented spots beside the eyes as $P_{b}$ , and the pigmented spots under the eyes as $P_{u}$ .

### 2.4 Extraction of wrinkles at the above inner corner of the eyes

We utilized OpenCV (Bradski, 2000) for general processing tasks for image files such as loading, saving, and segmentation. For facial landmark detection, dlib (Kazemi and Sullivan, 2014) was used. We measured the wrinkles above inner corner of the eyes, labeled $W_{a}$ , which has the thinnest epidermal thickness in the face relatively (Chopra et al., 2015). The area around the eyes was cropped as depicted in Figure 2b, using the coordinates of points in the eyebrow and eyelid lines. We performed the following image processing steps to extract wrinkles around the eyes. First, we converted the cropped images to grayscale. Next, we employed a ridge detection algorithm, the hybrid Hessian filter (Ng et al., 2015), to detect wrinkles in the grayscale images. Finally, we enhanced the wrinkles using the fast Fourier transform (Chung et al., 2011), which amplified the main signals and removed noise signals. The area detected as wrinkles was calculated as a wrinkle score, which was then normalized to a range of 0–100.

### 2.5 Calculation of morphological characteristics around the eyes

The morphological characteristics of the periorbital skin were calculated using the coordinates obtained from facial landmark detection. Morphological eye droopiness, labeled as $M_{d}$ , was defined as the height difference between the medial and lateral canthi. It was computed by taking the difference in the height of the medial canthus $H_{1}$ from the height of the lateral canthus $H_{2}$ , as illustrated in Figure 2c, using the following formula (Equation 1):

$$\text{Eye Droopiness }\left(M_{d}\right)=H_{1}-H_{2}$$ (1)

Temporal width, denoted as $M_{w}$ , was computed as the ratio of the sum of the distances from the outer corner of the eyes to the facial line ( $L_{2}+L_{3}$ ) to the facial width ( $L_{1}$ ). This normalization using facial width accounts for variations in overall size of the face. The calculation was based on the points indicated in Figure 2c and followed the formula (Equation 2):

$$\text{Temporal Width }\left(M_{w}\right)=\left(L_{2}+L_{3}\right)/L_{1}$$ (2)

The morphological periorbital features, $M_{d}$ and $M_{w}$ , can independently explain the sagging of the eyelid skin based on their respective x and y-coordinate. Notably, temporal hollowing, a common sign of aging, can impact the width of the facial temporal width (Nikolis et al., 2021; M et al., 2021). Similarly, the outer canthal distance, which serves as the counterpart to $M_{w}$ , decreases with age (Direk et al., 2016). To ensure consistency with the scales of other characteristics, two extracted morphological values were adjusted to ranges of 0–100, respectively.

### 2.6 Construction of age prediction model

We built age prediction models based on a distinct combination of periocular skin measurements. Each model was built based on linear regression method.

$$y=\beta_{0}+\sum \beta_{i}*x_{i}+\varepsilon$$

In the model, y represents the dependent variable (age), $\beta_{0}$ is the intercept, ε is the error, and $\beta_{i}$ is the beta coefficient of the *i*th independent variable, which corresponds to one of the measured skin characteristics. Based on this equation, we derived predicted age ( $\hat{y}$ ) (Figure 1). We built a total of nine different age prediction models and evaluated the performance of the combinations using 10-fold cross-validation method. In each model, 90% of the data out of 2,000 data were used to build the age prediction model, and the remaining 10% of the data were used to test the evaluation performance. Unlike typical predictive modeling studies that often select only the best-performing model, we developed multiple models using various combinations of skin features to suit diverse purposes. The final linear regression equation, also called the final model, was then extracted using the entire dataset. After then, to validate the final model, we used an additional independent validation dataset (n = 515) to calculate the validation performance. The predicted age calculated from the final equations were designated as the periorbital skin age.

### 2.7 Statistical analysis

Pearson correlation coefficient was used to quantify the performance as linear similarity between actual age and predicted age, in both evaluation and validation stages. It was also used to evaluate the linear correlation between chronological age and periorbital skin aging characteristics. To assess the clinical validity of periorbital skin age as a physiological biomarker, we analyzed its association with disease history using two approaches. First, we conducted logistic regression analysis to examine the association between periorbital skin age and the history of seven self-reported diseases, both individually and collectively. Statistical significance was controlled for multiple comparisons using the Benjamini-Hochberg false discovery rate (FDR) correction method. Second, we performed Principal Component Analysis (PCA) to reduce the dimensionality of the seven disease history variables and evaluated their correlation with periorbital skin age using Pearson correlation analysis of the first principal component (PC1). All statistical analyses were carried out using R software (version 4.3.0) (R Development Core Team, 2010), and the results were visualized using the ggplot2 package (Wickham, 2009).

## 3 Results

### 3.1 Changes in periorbital skin characteristics according to age

**FIGURE 3:**
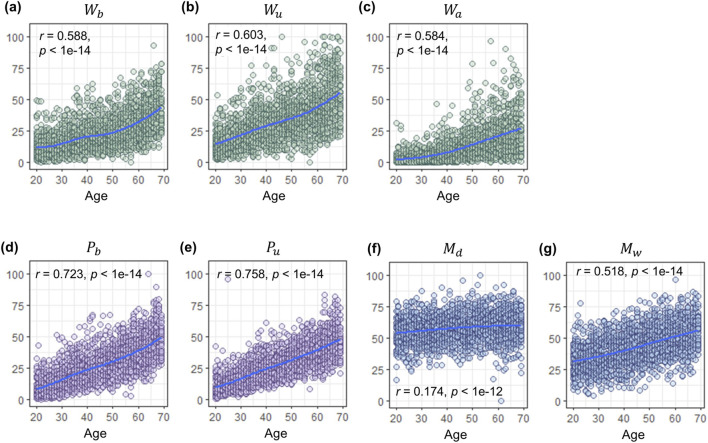
Age-related changes in periorbital skin characteristics. The y-axis represents the measured values of skin aging characteristics, and the x-axis represents actual age. The purple, green, and blue dots represent pigmented spots, wrinkles, and morphological features, respectively. The scatter plots show the age-related changes in **(a)** wrinkles beside the eyes ( $W_{b}$ ), **(b)** wrinkles under the eyes ( $W_{u}$ ), **(c)** wrinkles above the inner corner of the eyes ( $W_{a}$ ), **(d)** pigmented spots beside the eyes ( $P_{b}$ ), **(e)** pigmented spots under the eyes ( $P_{u}$ ), **(f)** eye drooping ( $M_{d}$ ), **(g)** temporal width ( $M_{w}$ ). The correlation with age is shown as *r*, with corresponding *P*-value as *p*.

We extracted seven aging indices belonging to the three categories, including wrinkles, morphological features, and pigmented spots (Figure 2). As depicted in Figures 3a–g, clear increasing trends of all the seven aging indices with age were investigated. The correlation coefficients of the three categories showed (1) the highest values for two indices of pigmented spots, $P_{b}$ and $P_{u}$ (0.723 and 0.758, respectively), (2) moderate values for three indices of wrinkles, $W_{b}$ , $W_{u}$ , and $W_{a}$ (0.588, 0.603, and 0.584, respectively), and (3) the lowest values for two indices of the periorbital morphology, $M_{d}$ and $M_{w}$ (0.174 and 0.518, respectively). It is notable that $M_{w}$ exhibited approximately three times stronger correlation with age and steeper increase than $M_{d}$ .

### 3.2 Age prediction models based on periorbital skin characteristics

As shown in Table 1, nine age prediction models were established based on the varied combinations of seven aging indices. Model 1-3 were built using only one category each (wrinkles for model 1, morphological features for model 2, and pigmented spots for model 3). Models 4-6 were created by pairs of two from three categories, and model 7 covered all categories. To use only device-based features using the Janus-III system, models 8 and 9 were modified by removing the variable $W_{a}$ from models 1 and 5, respectively. As a result, models 3, 8, and 9 were exclusively constructed based on the metrics obtained from the Janus-III system.

**TABLE 1:** Construction of age prediction models and evaluation of performance.

Model No.	Wrinkle			Morphology		Pigmented spot		Performance	
	$W_{b}$	${\;W}_{u}$	$W_{a}$	$M_{d}$	$M_{w}$	$P_{b}$	${\;P}_{u}$	Evaluation	Validation
1	O	O	O	​		​		0.702	0.705
2	​			O	O	​		0.524	0.593
3	​			​	​	O	O	0.784	0.786
4	O	O	O	O	O	​		0.760	0.778
5	O	O	O	​		O	O	0.802	0.804
6	​			O	O	O	O	0.808	0.822
7	O	O	O	O	O	O	O	0.822	0.834
8	O	O	​	​		​		0.669	0.681
9	O	O	​	​		O	O	0.787	0.793

‘O’ symbols denote the model components composing the models. Performance was measured by the correlation coefficient between the predicted and actual ages. $W_{b}$ , Wrinkles beside the eyes; $W_{u}$ , wrinkles under the eyes; $W_{a}$ , wrinkles above the inner corner of the eyes; $M_{d}$ , eye drooping; $M_{w}$ , temporal width; $P_{b}$ , pigmented spots beside the eyes; $P_{u}$ , pigmented spots under the eyes.

The performance of the established models was evaluated using the correlation coefficient between predicted and actual ages. Each model underwent a 10-fold cross-validation process, with results displayed as correlation coefficients of evaluation in Table 1. Except for the model 2 and 8, most of the models showed high performance greater than 0.7. Notably, the model 3, 5, 6, 7, and 8, which adopted the two pigmentation factors $P_{b}$ and $P_{u}$ , showed higher performance than the best-performing model of the others (*r* of model 4 = 0.760).

### 3.3 Refinement and validation of periorbital age prediction models

After evaluating model performance, we derived final equations for each model using the complete dataset (Supplementary Table S1). Among the nine models, model 5, 7, and 9 exhibited negative beta coefficients due to collinearity among factors. To create more intuitive and concise models, we modified Equations 5, 7, 9 by removing variables with negative beta coefficients. These modified versions are denoted with double prime symbols (˝) in Supplementary Table S1. To validate the final models, we applied them to an independent test set (n = 515). The validation coefficients were calculated by comparing between actual and predicted ages (Table 1; Supplementary Table S2). These equations can be used directly to generate periorbital skin age based on measured periorbital skin features and to predict aging.

### 3.4 Association study between periorbital skin age and medical history

We investigated periorbital skin age as a potential biomarker of individual health profile. Using model 7, which aggregates all periorbital skin aging characteristics, we found significant associations between periorbital skin age and the presence of at least one disease in our test set of 515 subjects (Table 2). Of the seven disease histories examined, five (from D2 to D6 in Table 2) showed significant associations with the periorbital skin age (FDR <0.05). PCA result of disease history data revealed that PC1 exhibited consistent directional patterns across all diseases (Supplementary Figure S1), and demonstrated a significant correlation with the periorbital skin age (*r* = 0.39). These findings suggest that the periorbital skin age effectively reflects medical background. Notably, the association between periorbital skin age and the presence of at least one disease remained significant even after adjusting for chronological age ( β = 0.03, *P*-value = 0.038).

**TABLE 2:** The associations of disease history with actual age and with eye aging index.

Code	Disease^a^	Age		Eye aging index		
		β	*P*-value	β	*P*-value	FDR
D0	History of any following diseases	0.10	**<2e-16**	0.09	<**2e-16**	**<2e-15**
D1	Heart diseases	0.01	0.505	0.01	0.656	0.656
D2	High blood pressure	0.16	**0.016**	0.03	**0.003**	**0.005**
D3	Hyperlipidemia	0.12	**2e-11**	0.07	**3e-09**	**8e-09**
D4	Fatty liver	0.10	**9e-06**	0.07	**2e-05**	**4e-05**
D5	Kidney diseases	0.10	**5e-13**	0.07	**2e-11**	**8e-11**
D6	Diabetes	0.05	**0.011**	0.04	**0.012**	**0.016**
D7	Cancer	0.02	0.404	0.02	0.443	0.506

^a^ Self-reported disease history. Bold indicates values with P-value < 0.05 or FDR < 0.05. β: beta coefficient; FDR: false discovery rate.

Additional association tests were conducted to assess lifestyle factors, including sun exposure duration, frequency of sunscreen application, and smoking status, in relation to periorbital skin age. The results indicated that prolonged sun exposure duration, reduced frequency of sunscreen use, and smoking were associated with increased periorbital skin age, however, only sun exposure duration reached statistical significance. When testing the association between the presence of at least one disease and periorbital skin age, with adjustment for both chronological age and sun exposure duration, the association was attenuated and no longer statistically significant ( β = 0.02, *P*-value = 0.101).

## 4 Discussion

Previous studies have employed various methods to assess periorbital skin aging, including morphological aging, wrinkles, photoaging, and pigmented spots around the eyes (Liu et al., 2023; Odunze et al., 2008; Fitzpatrick et al., 1996; Oh et al., 2023). While these established methods can provide detailed information on specific signs of skin aging, they often focus on each phenotypic trait, making it difficult to conduct a comprehensive assessment of skin aging. In this study, we developed models that combine multiple periorbital skin aging characteristics into a single, unified indicator for a more inclusive evaluation of periorbital skin aging.

We measured seven periorbital skin aging characteristics using a skin measurement device and advanced computational techniques for facial image analysis, focusing on wrinkles, morphology, and pigmented spots in periorbital region in a total of 2,515 Korean women. We integrated these seven characteristics into a linear regression model with weights for each characteristic to create an age prediction model that derives a single periorbital skin age, serving as an indicator of eye skin longevity. The periorbital skin age provides a more efficient way to compare the condition of the periorbital skin before and after treatment, while also allowing for inter-individual comparisons.

Our analysis revealed significant age-related correlations with wrinkles and pigmented spots around the eyes (Figures 3a–e). Cumulative changes in facial musculature and repetitive expressions initially produce transient wrinkles, eventually leading to permanent ones. Notably, periorbital wrinkles exhibited a more rapid age-related increase in depth compared to other facial areas (Takema et al., 1997). Photoaging plays a crucial role in this process by enhancing matrix metalloproteinase transcription, thereby reducing collagen synthesis and promoting wrinkle formation (Varani et al., 2001; Moloney et al., 1992). It also induces persistent melanocyte activation, resulting in hyperpigmentation (Kim et al., 2022). While genetic factors may influence periorbital pigmentation, studies indicate an age-associated increase as well (Goodman and Belcher, 1969; Suppa et al., 2011), suggesting a multifactorial etiology for age-related periorbital skin changes.

We investigated the morphological aging of the periorbital area and observed significant associations between age and both eye droopiness and temporal width (Figures 3f,g). A previous study demonstrated an increase in drooping of the upper eyelid with age (Flament et al., 2018). Similarly, our study found age-related drooping in the lateral canthus (outer corner of the eye). The results imply that the loss of skin tension around the eyes can be expressed differentially according to the skin area. In addition, skeletal structural changes and muscle atrophy due to aging can affect skin morphological characteristics. The increase in temporal width with age can be affected by changes in the bony orbit around the eyes and the orbicularis oculi muscle. Specifically, the attachment of the orbicularis oculi muscle to the orbital rim gradually decreases (Okuda et al., 2023).

Notably, the biological pathways of skin aging are linked to various aging-related diseases (Chin et al., 2023). We investigated whether periorbital skin aging could indicate an individual’s medical history, as this area effectively represents overall skin aging, the most visible manifestation of aging (Farage et al., 2013). We found significant associations between the calculated periorbital skin age and five out of seven diseases. All examined diseases have been previously reported to be associated with aging (Berben et al., 2021; Kirkman et al., 2012; Fang et al., 2020; Rosada et al., 2020; Cheng et al., 2022; Lakatta and Levy, 2003). To evaluate the utility of periorbital skin age as a metric distinct from chronological age, we examined the association between disease history and periorbital skin age after adjusting for chronological age. We found that disease history (D0 in Table 2) remained significantly associated with periorbital skin age even after this adjustment. This indicates that, within the same age group, individuals with higher periorbital skin age have a greater prevalence of disease history.

While we adjusted for chronological age, other influential factors such as sun exposure duration, smoking, and skincare routines were not fully controlled. Additional analyses revealed a significant association between sun exposure duration and periorbital skin age. When adjusting for both chronological age and sun exposure, the association with disease history was attenuated, suggesting potential mediation or confounding effects. This suggests that environmental factors partly explain the link between skin aging and disease prevalence. Further research with more comprehensive lifestyle and environmental data will be essential to clarify these complex interactions and enhance the predictive value of this index.

It is important to acknowledge that the disease categories used in this study were broadly defined and based on self-reported data. Although definitions for each disease category were provided in the Materials and Methods section, potential biases such as recall bias or under-reporting may affect the accuracy of disease history. These factors should be taken into account when interpreting our findings, and future studies incorporating clinically verified diagnoses are needed to validate and strengthen these associations.

In this study, we developed periorbital skin age as a biomarker for assessing skin aging and disease history using suggested age prediction models. These models can be applied adaptively and are useful when specific skin characteristics are inaccessible for measurement. For a comprehensive periorbital aging assessment, model 7 incorporating all three categories is the most suitable, while for evaluating skin-tightening cosmetic treatments, a model focusing only on wrinkles and morphology will be appropriate. Our approach utilizes traditional signs of aging like wrinkles and pigmented spots, while also taking into account morphological changes that have not typically been considered in previous studies.

While these findings are promising, this study has some limitations, including its population specificity to Korean women and the cross-sectional design, which limits causal inference. The observed association between periorbital skin age and medical history likely reflects shared biological pathways, such as chronic inflammation, oxidative stress, and cellular senescence that link skin aging with systemic diseases (Franco et al., 2025; Baumer et al., 2018; Gelfand et al., 2006; Thau et al., 2025). To better understand these mechanisms and establish causality, longitudinal studies with more diverse cohorts, including different ethnicities and sexes, are needed. Future research incorporating broader populations and extended follow-up will be essential to validate and expand upon our results.

By incorporating these periorbital aging features into a single index, our models offer an effective method for evaluating treatments aimed at improving eye skin aging, assessing individual medical profiles, and gaining extensive understanding of the periorbital skin area.

## Data Availability

The raw data supporting the conclusions of this article will be made available by the authors, without undue reservation.
