# Supplementary material for: Periorbital skin index as a biomarker for biological aging and health status
Source: Front Aging. 2026 Jan 29;7:1715245. doi: 10.3389/fragi.2026.1715245 (PMC12894317; doi:10.3389/fragi.2026.1715245)
Supplement: Supplementary file 1 [file Supplementaryfile1.docx]

**Supplementary Table 1. Equations of final age prediction models**

| **No.** | **Model** |
| --- | --- |
| 1 | $27.71+0.30*W_{b}+0.20*W_{u}+0.29*W_{a}$ |
| 2 | $16.83+0.11*M_{d}+0.50*M_{t}$ |
| 3 | $22.91+0.30*P_{b}+{0.49*P}_{u}$ |
| 4 | $14.64+0.26*W_{b}+0.16*W_{u}+0.88*W_{a}+0.04*M_{d}+0.31*M_{t}$ |
| 5 | $23.66-0.07*W_{b}+0.01*W_{u}+0.22*W_{a}+0.31*P_{b}+0.40*P_{u}$ |
| 5**˝** | $23.53+0.79*W_{a}+0.27*P_{b}+0.40*P_{u}$ |
| 6 | $13.45+0.05*M_{d}+0.21*M_{t}+0.27*P_{b}+0.42*P_{u}$ |
| 7 | $15.02-0.04*W_{b}+0.01*W_{u}+0.20*W_{a}+0.04*M_{d}+0.20*M_{t}+0.27*P_{b}+0.33*P_{u}$ |
| 7**˝** | $14.93+0.01*W_{u}+0.20*W_{a}+0.04*M_{d}+0.20*M_{t}+0.25*P_{b}+0.33*P_{u}$ |
| 8 | $26.48+0.35*W_{b}+0.30*W_{u}$ |
| 9 | $22.53-0.05*W_{b}+0.08*W_{u}+0.33*P_{b}+0.42*P_{u}$ |
| 9**˝** | $22.46+0.07*W_{u}+0.30*P_{b}+0.42*P_{u}$ |

Beta coefficients and performance of each model were depicted. Models containing negative beta coefficients were recalculated after removing these elements.

**Supplementary Table 2. Overview of structure and statistics of all age prediction models**

| Model  No. | Wrinkle | | | Morphology | | Pigmented spot | | Performance | |
| --- | --- | --- | --- | --- | --- | --- | --- | --- | --- |
|  | $W_{b}$ | $W_{u}$ | $W_{a}$ | $M_{d}$ | $M_{t}$ | $P_{b}$ | $P_{u}$ | Evaluation | Validation |
| 1 | O | O | O |  | |  | | 0.702 | 0.705 |
| 2 |  | | | O | O |  | | 0.524 | 0.593 |
| 3 |  | | |  |  | O | O | 0.784 | 0.786 |
| 4 | O | O | O | O | O |  | | 0.760 | 0.778 |
| 5 | O | O | O |  | | O | O | 0.802 | 0.804 |
| 5**˝** | ― | ― | O |  | | O | O | ― | 0.801 |
| 6 |  | | | O | O | O | O | 0.808 | 0.822 |
| 7 | O | O | O | O | O | O | O | 0.822 | 0.834 |
| 7**˝** | ― | O | O | O | O | O | O | ― | 0.833 |
| 8 | O | O |  |  | |  | | 0.669 | 0.681 |
| 9 | O | O |  |  | | O | O | 0.787 | 0.793 |
| 9**˝** | ― | O |  |  | | O | O | ― | 0.791 |

‘O’ symbols denote the model components composing the models, and double prime marks (˝) indicate modified versions of the original model. Elements represented by the dash (―) were excluded from the final equation due to their negative beta coefficients.


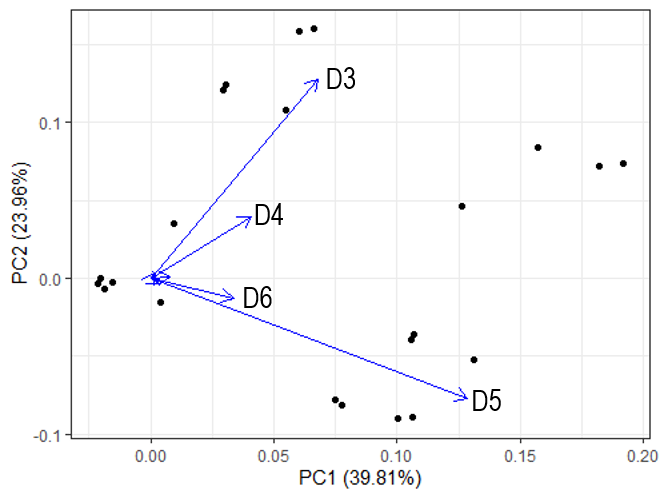


**Supplementary Figure 1. Results of principal component analysis**

Principal component analysis (PCA) of disease histories. Blue arrows represent loadings of each disease history (D3-D6), with arrow length indicating the strength of contribution. The first principal component (PC1) explains 39.81% of total variance, and the second principal component (PC2) accounts for 23.96%. Only D3 to D6 are shown as vectors due to their strong contributions to the principal components.
